# Soy Protein Hydrolysates Affect the Structural and Mechanical Properties of Soy Protein-Wheat Gluten Extrudates Using High Moisture Extrusion

**DOI:** 10.3390/foods12050912

**Published:** 2023-02-21

**Authors:** Yan Ji, Zhaojun Wang, Qian Deng, Jie Chen, Zhiyong He, Maomao Zeng, Fang Qin, Hongyang Pan

**Affiliations:** 1 State Key Laboratory of Food Science and Technology, Jiangnan University, Wuxi 214122, China; 2 International Joint Laboratory on Food Safety, Jiangnan University, Wuxi 214122, China

**Keywords:** hydrolyzed soy protein isolate, high moisture extrusion, plasticizer, texture properties, rheological properties

## Abstract

This study aimed to investigate the effect of hydrolyzed soy protein isolate (HSPI) as a plasticizer in the soy protein mixture-wheat gluten (SP-WG) extrudates on its structural and mechanical properties during high moisture extrusion. Those SP were prepared by mixing soy protein isolate (SPI) and HSPI with different ratios. HSPI primarily consisted of small molecular weight peptides measured with size exclusion chromatography and sodium dodecyl sulfate-polyacrylamide gel electrophoresis. The elastic modulus of SP-WG blends decreased with increased HSPI contents through the closed cavity rheometer. Adding HSPI at low concentrations (≤30 wt% of SP) enhanced a fibrous appearance and higher mechanical anisotropy while adding more HSPI resulted in a compact and brittle structure and tended to be isotropic. It can be concluded that the partial addition of HSPI as a plasticizer can promote the formation of a fibrous structure with enhanced mechanical anisotropy.

## 1. Introduction

Currently, the reduction of animal protein consumption is promoted due to agronomic, ecological, public health, and ethical issues. Plant proteins have become a sustainable and healthy option and can supply us with an extensive diversity of sources [1,2]. Extrusion technology (low moisture 20–40% and high moisture 40–70%) is currently the most employed and established technique to produce fibrous products starting from various protein-enriched ingredients or mixtures [3,4]. The high moisture extrusion technology is regarded as a viable choice for developing meat analogs because it possesses the advantages of high efficiency, lower energy input, no waste discharge, and pronounced fibrous structure [5].

A fibrous structure can be achieved from an aqueous dispersion of two thermodynamically incompatible phases that consist of either two proteins or a protein with polysaccharides when the dispersed phase is distorted and aligned by flow [6,7,8,9,10,11]. For instance, blends of soy protein isolate (SPI) and wheat gluten (WG) were particularly used to produce fibrous macrostructure, of which the WG forms a continuous network enrobing elongated SPI domains [9]. Schreuders et al. (2020) found that SPI-WG is possibly an intermediate system between a bi-continuous structure and a system with a stronger dispersed phase. Similarities in the rheological properties of the phases are necessary for such simultaneous deformation, and the phase volumes and spatial distribution of the phases are regulated by the water distribution between the phases [12]. More water was taken in by the SPI phase than by the WG phase, which resulted in the two phases having identical rheological qualities in most blends. [7]. Moreover, Cheftel et al. (1992) discovered layers or coarse fibers arranged in the direction of flow through the die create an anisotropic structure in SPI containing 5–10% wheat gluten [13]. Zhang et al. (2022) found that a blend of soy protein concentrate-wheat gluten at 50/50 resulted in the best fibrous structures [14]. Chiang et al. (2019) reported that soy protein containing 30% WG could be the closest in terms of structural properties compared with boiled chicken breast [15]. Thus, SPI and WG were chosen as raw materials for high moisture extrusion in this study.

During extrusion, polymer chains are denaturized, dissociated, unraveled, and aligned to create a homogeneous protein melt. Covalent cross-links are detrimental because they reduce chain mobility, raise viscosity, and prevent homogenization. The addition of plasticizers to proteins enables to tailor of the mechanical and thermal properties of extrudates by increasing molecular mobility and decreasing viscosity [16,17,18,19,20,21,22]. Hydrophilic compounds (e.g., glycerol and amines) [20,23] and amphiphilic plasticizers (e.g., fatty acids and some surfactants) [19,24] are commonly used to reduce their processing temperature. Recently, low molecular mass proteins or protein hydrolysates received increasing attention as a plasticizer due to their compatibility with proteins. Protein hydrolysates contain small peptides and free amino acids, which possess antioxidative and antimicrobial activities. Hydrolysates have been used in extrusion to improve the texture and nutritional profile of products. Hoyos-Concha et al. (2022) found that adding high-protein hydrolyzed decreased the specific mechanical energy (SME) during extrusion due to the increased mass flow. The resulting extruded feed had increased durability and decreased hardness [25]. A similar result was found by Suratkar (2016) that adding hydrolyzed bloodmeal produced extrudate with lower SME. The tensile strength, energy at the break, and glass transition temperature (Tg) decreased with increasing the number of hydrolysates [26]. Tuck et al. (2014) showed wheat gluten could be plasticized with its own tryptic hydrolysate, and the peptides have an effect on the WG analogous to that of glycerol, which can decrease the modulus and Tg [27]. According to Nuanmano et al. (2015), the highly mobile short-chain peptide of gelatin hydrolysate may function as a plasticizer in protein-based films, notably those derived from fish myofibrillar protein. Incorporating gelatin hydrolysate has the potential to reduce interaction between chains of proteins and enhance the free volume in the protein network of the film [28]. Oterhals & Samuelsen (2015) found that water-soluble compounds in fishmeal influence glass and flow transition temperatures [29]. Thus, low molecular mass and compatible protein are required to improve processability and final material characteristics by decreasing macromolecular associations during extrusion.

This study examines the plasticizing effects of hydrolyzed soy protein isolate (HSPI) in SP-WG extrudates. Solubility, water holding capacity, sodium dodecyl sulfate-polyacrylamide gel electrophoresis, and molecular weight distribution of SPI and HSPI were measured. The viscoelastic properties of SP-WG blends in water were determined by a time sweep test and a temperature sweep test. The extrudates were prepared in an extruder using blends consisting of 30 wt% WG and 70 wt% SP with varying concentrations of HSPI. The mechanical and structural properties of the extrudates were determined with a texture analyzer, confocal scanning laser microscopy (CSLM), and a scanner. Moreover, the bulk density and permeability of the extrudates were also measured.

## 2. Materials and Methods

### 2.1. Materials

Soy Protein isolate (SPI, SD-100) was purchased from Linyi Shansong Biological Products Co., Ltd. (Linyi, China). SPI powder contains 90.0 wt% protein (dry base), 7.0 wt% moisture, 0.8 wt% fat, and 2 wt% carbohydrates. Commercial hydrolyzed soy protein isolate (HSPI, 791) was purchased from Suizhong Song Zhiyuan Plant protein Co., Ltd. (Huludao, China). The HSPI was enzymatically hydrolyzed protein, according to the manufacturer’s specifications. The product contains 90.5 wt% protein (dry base) and 6.2 wt% moisture. Wheat Gluten (WG) was purchased from Zhongyu Food Co., Ltd. (Binzhou, China) with a moisture content of 7.7 wt%. WG contains 85.6 wt% protein, 0.9 wt% ash, and 0.8 wt% fat on a dry matter basis, according to the manufacturer’s specifications. All other chemicals were of analytical grade unless otherwise stated. Throughout the experiments, deionized water was used to prepare aqueous solutions.

### 2.2. Nitrogen Solubility Index and Water Holding Capacity

The nitrogen solubility index (NSI) and water holding capacity (WHC) of SPI and HSPI was obtained using a 2 wt% dispersion. Before centrifugation at 10,000× *g* for 30 min at 20 °C, the dispersions were properly mixed and agitated overnight at 25 °C, and the supernatant and pellet were separated and recorded. Subsequently, the supernatant was used to determine the soluble protein content using the Biuret method at 540 nm. The Kjeldhal method was used to measure the total protein content of the powder (N × 6.25). Hydrolysates’ NSI was determined by dividing the soluble protein content of the supernatant by the total protein content of the initial powder in the sample. The pellet was used to determine WHC, that is, the mass of absorbed water per gram of protein.

### 2.3. Sodium Dodecyl Sulfate-Polyacrylamide Gel Electrophoresis

Sodium dodecyl sulfate-polyacrylamide gel electrophoresis (SDS-PAGE) was performed according to the method reported by Liu et al. (2000) with slight modifications [30]. On a discontinuous buffered system, SDS-PAGE was performed using a separating gel of 12% and a stacking gel of 4%. Samples (4 mg/mL) were diluted (1:1, *v/v*) with and without β-mercaptoethanol (5%, *v/v*) in a buffer containing 0.0625 M Tris-HCl, 10% glycerine, 2% SDS, and 0.0025% bromophenol blue. All samples were then boiled in a water bath for 5 min. 15μL of the prepared samples were loaded onto the gels. The electrophoresis was run at 80 V and then at 120 V. The eluted gels were photographed in a gel imaging system (version 5.2; 2104 Bio-Rad Laboratories, Irvine, CA, USA).

### 2.4. Molecule Weight Distribution

The molecule weight distribution was determined by high-performance liquid chromatography (HPLC) using HPLC equipment (Waters 2695, Milford, MA, USA) with a Shodex protein KW-804 column (Shodex Co., Tokyo, Japan). Deionized water was used to dilute the SPI and HSPI dispersion to 10 mg/mL before being filtered through 0.45 m film. The elution buffer was a phosphate buffer with a concentration of 0.05 mol/L (pH 7.0, containing 0.03 mol/L sodium chloride. A flow rate of 1 mL/min was used during the elution process. The analysis used a 10 μL injection volume. The detection wavelength was 280 nm. Bovine thyroglobulin (MW 669 kDa), bovine serum albumin (MW 67 kDa), ovalbumin (MW 43 kDa), phosphatase (MW 32 kDa), and aprotinin (MW 6.5 kDa) were molecular weight (MW) standards. The calibration curve was shown below: y = −0.5577lgMW + 10.458 (where y is the retention time of the standard).

### 2.5. Rheological Measurements

A closed cavity rheometer (RPA Elite, TA Instruments, New Castle, DE, USA) was used to assess the reactions of SP -WG blends under different thermal and mechanical stresses [7,31]. The soy protein mixtures were prepared by mixing SPI and HSPI with weight ratios of 10:0, 8:2, 7:3, 5:5, 3:7, 2:8, 0:10, which were named SPI:HSPI = 10:0, SPI:HSPI = 8:2, SPI:HSPI = 7:3, SPI:HSPI = 5:5, SPI:HSPI = 3:7, and SPI:HSPI = 0:10, respectively. These soy protein mixtures were mixed with WG at a ratio of 7:3. A 45 wt% SPI: HSPI-WG mixture was prepared and hydrated at 4 °C for at least 24 h. Approximately 5 g was placed in a closed chamber between two plastic films. Sealing the cones prevents water evaporation at high temperatures so that a cavity pressure of 4.5 MPa can be achieved. The bottom cone oscillated to give mechanical treatment, and the angular frequency ω and strain γ can be modified to meet individual needs. First, a 15-min oscillation time sweep experiment was conducted at a high frequency (10 Hz) and strain (80%) at temperatures of (100, 110, 120, 130, 140 °C). Second, a temperature sweep experiment (10 Hz, 80%) was conducted at a heating rate of 5 K/min from 40 °C to 140 °C.

### 2.6. Extrusion Process and Preparation

Our preliminary trials were performed with different ratios of SPI and WG from 10:0 to 0:10 and found that the SPI-WG extrudate with a ratio of 7:3 had a pronounced fibrous appearance and the highest mechanical anisotropy. Thus, the test sample consisting of soy protein mixtures (SP) and WG at a ratio of 7:3 was prepared the same as in a closed cavity rheometer based on our preliminary trials [15]. An extruder with a twin-screw, co-rotating design (FMHE-36, FUMACH, Changshang, China) with a 36:1 length-to-diameter ratio was used to prepare the extrudate. There are 8 zones on the barrel of the extruder that can be separately heated and thermally controlled. The SP-WG mixtures were fed into the extruder at a constant rate of 8.0 kg/h (dry basis). Water was introduced into the extruder by a pump. Throughout the trials, the screw configuration consisted of forward and reverse transport elements. The extrusion conditions were fixed at a feed moisture content of 55 wt% (dry basis), the screw speed of 280 rpm, and the extruder barrel temperatures of 60 °C, 80 °C, 110 °C, 130 °C, 140 °C, 140 °C, and 110 °C from the second zone to the eighth, respectively. The end of the extruder was fitted with a cooling die (dimensions, 10 × 70 × 926 mm, H × W × L). Running water kept the die at 70 °C. During steady-state operating conditions, extrudates were collected and were named SPI:HSPI = 10:0 + WG, SPI:HSPI = 8:2 + WG, SPI:HSPI = 7:3 + WG, SPI:HSPI = 5:5 + WG, SPI:HSPI = 3:7 + WG, SPI:HSPI = 2:8 + WG, and SPI:HSPI = 0:10 + WG. Except for visual observation and textural analysis, all extrudates were vacuum packaged and then stored in a sealed bag in a freezer (−18 °C) before further analysis.

### 2.7. Macro- and Microstructures of Extrudates

#### 2.7.1. Visual Observations

To visualize the fibrousness on a macroscopic scale, the morphologies of fresh SP-WG extrudates were analyzed by manually deforming the samples. All the materials were taken near the edge (10–20 mm from the center) for a fair comparison.

#### 2.7.2. Confocal Scanning Laser Microscopy

A confocal laser scanning microscope was used to visualize the microstructures of the extrudates. The samples were cut in parallel and perpendicular to the direction of extrusion (approximately 20 mm) and subsequently pre-frozen rapidly with a freeze embedding agent (Tissue-Tek O.C.T Compound, Sakura Finetek, Torrance, CA, USA) at −80 °C. Samples were placed on a tray, sectioned with a cryo-microtome (CM1950, Leica, Germany) at −20 °C, sliced approximately 20 μm thick in a regular pattern, affixed to a pretreated slide, and stored at −20 °C in the refrigerator. The proteins were stained with Rhodamine B at a concentration of 0.2 mg/mL. Visualization was carried out with a confocal scanning laser microscope type TCS SP8 (Leica, Wetzlar, Germany) using an OSPL laser at 552 nm. Images were captured with an HC PL APO 10×/0.40 CS lens. All Images were captured with a resolution of 1024 × 1024 pixels and processed using LAS AF software (2.6.3, Leica microsystems, Wetzlar, Germany).

#### 2.7.3. Scan Images

A scanner (M125126, HP Co., Palo Alto, CA, USA) was used to record the scanned images of the extrudates [32]. The extrudate slices prepared from 2.7.2 were scanned to produce the scanned images. The image had a 1200-pixel-per-inch resolution.

### 2.8. Textural Properties

The textural properties of fresh extrudates were evaluated by measuring tensile strength with a TA.XT2 Texture Analyzer (Stable Micro Systems, Ltd., Godalming, UK) at room temperature. A static load cell with a force of 100 N was used for the texture analyzer. A dog bone-shaped mold was used during the tensile test, which resulted in the samples being cut parallel or perpendicular to the extrusion direction. The samples were approximately 15.2 mm in length and 3.18 mm in width and varied in thickness between 4 and 6 mm. The tensile tests were measured with an A/TG probe at a constant displacement speed of 3.0 mm/s. Before the measurement, the samples were placed between the fixtures at a distance of 15.2 mm. The abrasive paper was used to prevent slipping during testing. A stress–strain curve was used to determine tensile stress and tensile strain at rupture. On the stress–strain curve, the fracture point is the point where there is a dramatic decrease in stress. Force–displacement curves are presented as the results of these tensile test analyses. The true stress (*σ*, Pa) and fracture strain (*ε*, −) are defined as follows:

(1) $$\varepsilon_{h}=\ln\frac{h\left(t\right)}{h_{0}}\;\left[-\right]$$

(2) $$A\left(t\right)=\frac{h\left(t\right)}{h_{0}}\;\cdot \;A_{0}\;\left[m^{2}\right]$$

(3) $$\sigma \left(t\right)=\frac{F\left(t\right)}{A\left(t\right)}\;\left[\mathrm{Pa}\right]\#$$

where *h*_0_ is the length of the sample (e.g., 15.2 mm), *h*(*t*) is the length over time t, *A*(*t*) is the cross-sectional area of the bar, *A*_0_ is the area of the initial contact surface, and *F*(*t*) is the force per unit area of *A*(*t*). Nine specimens in parallel and perpendicular directions were collected for every sample, respectively.

### 2.9. Statistical Analysis

All measurements were carried out in triplicate unless otherwise specified. Analysis of variance (ANOVA) was used to analyze the characteristics of SPI and HSPI. The LSD test with a 95% confidence interval was applied in the calculations, which were performed using Statisticix version 9.0 (Analytical Software, Talla-hassee, FL, USA). The significance level of the results was set at *p* < 0.05.

## 3. Results and Discussion

### 3.1. Physicochemical Properties of SPI and HSPI

#### 3.1.1. Nitrogen Solubility Index and Water Holding Capacity

**Figure 1:**
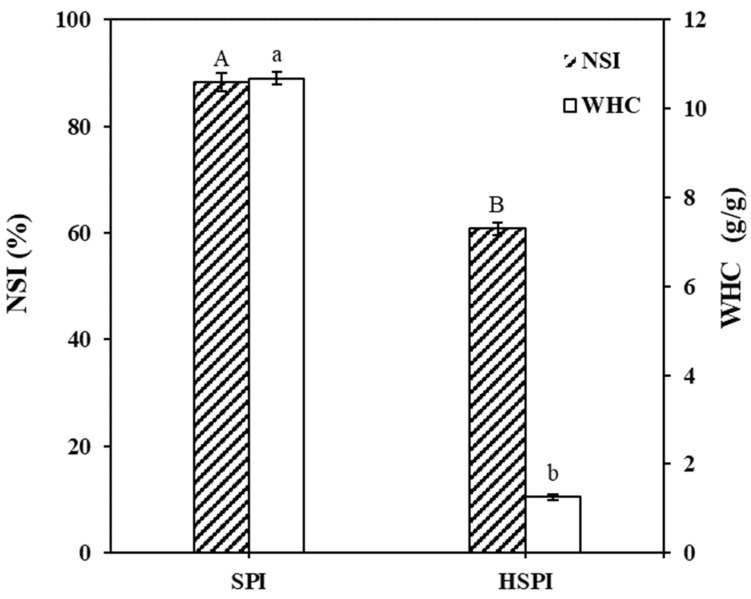
Nitrogen soluble index (NSI) and water holding capacity (WHC) of SPI (soy protein isolate) and HSPI (hydrolyzed soy protein isolate). Significant differences are indicated by different lowercase letters (A-B, a-b) (*p* < 0.05).

The hydration properties of SPI and HSPI were compared using the nitrogen solubility index (NSI) and water holding capacity (WHC) (Figure 1). The commercial HSPI had a lower NSI (60.75%) than the SPI (88.25%). This is probably due to the hydrophobic and disulfide bond aggregation between the hydrolysates [33]. Furthermore, the WHC of HSPI (1.25 g/g) was much lower than that of SPI (10.68 g/g). This is because small-molecular-weight peptides in HSPI cannot hold water [34].

#### 3.1.2. Molecular Weight Distribution

**Figure 2:**
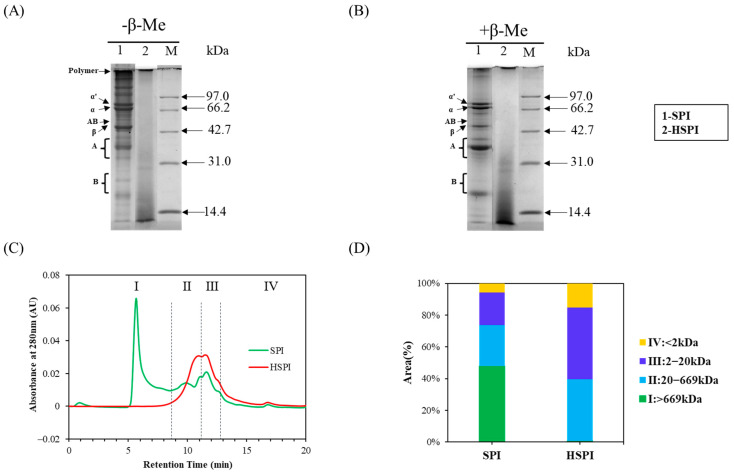
Non-reduced (**A**) and reduced pattern (**B**) SDS-PAGE profiles of SPI (Lane 1) and HSPI (Lane 2). M: molecular weight marker (kDa), β-Conglycinin: α’ (86 kDa), α (66 kDa), and β (51 kDa); Glycinin: (A), acidic subunit (34–43 kDa), and (B), basic subunit (17–26 kDa); (**C**): Size exclusion chromatography (SEC) profiles of SPI and HSPI. The Roman numerals indicated different zones: zone I, >669.0 kDa; zone II, 20.0–669.0 kDa; zone III, 2.0–20.0 kDa; zone IV, <2.0 kDa. (**D**): Individual zone integrated areas of chromatogram peaks for SEC profiles of SPI and HSPI.

SDS-PAGE was performed in the absence and presence of β-mercaptoethanol (Figure 2A,B) to track the variation of protein subunits. Two major factions of soy protein were namely 11S globulin (glycinin; A and B are short for acidic and basic polypeptides, respectively) and 7S globulin (β-conglycinin; α’, α, and β). For HSPI, the band intensities of aggregates, α’, α, and β and AB vanished, and the band intensities of polypeptides (Mw < 14.4 kDa) increased. Furthermore, after the addition of β-mercaptoethanol, the band on the top of the gel was still notable for both SPI and HSPI (Figure 2B), indicating that some non-covalent bonds also contributed to the protein aggregates (Mw > 100 kDa) apart from disulfide covalent bonds.

Size exclusion chromatography (SEC) was used to study further the molecular weight distribution of SPI and HSPI (Figure 2C,D). The SEC profile of SPI and HSPI can be divided into four zones according to the distributions of chromatogram peaks. They are zone I: <8.303 min (Mw > 669.0 kDa), zone II: 8.303–11.039 min (Mw, 20.0–669.0 kDa), zone III: 11.039–12.835 min (Mw, 2.0–20.0 kDa), and zone IV: >12.835 min (Mw < 2.0 kDa), corresponding to large protein aggregates, a mixture of 7S (150–190 kDa), 11S (320–360 kDa) and 15S (dimer of glycinin), a mixture of 2S (<20 kDa) and subunits of 7S and 11S, and small-molecular-weight peptides, respectively [33,35,36]. About 48% of the soluble protein in SPI was large protein aggregates while approximately 60% of soluble protein of HSPI was distributed in Mw < 20.0 kDa, which was in accordance with the SDS-PAGE result (Figure 2A,B).

### 3.2. Rheological Properties

#### 3.2.1. Time Sweep of SP-WG Blends

**Figure 3:**
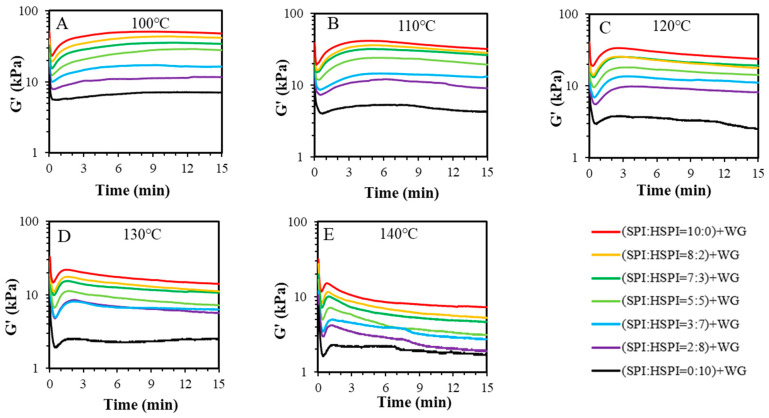
Time sweep measurements (80% strain, 10 Hz frequency) for SPI:HSPI = 10:0 + WG, SPI:HSPI = 8:2 + WG, SPI:HSPI = 7:3 + WG, SPI:HSPI = 5:5 + WG, SPI:HSPI = 3:7 + WG, SPI:HSPI = 2:8 + WG, and SPI:HSPI = 0:10 + WG at various temperatures: (**A**) 100 °C, (**B**) 110 °C, (**C**) 120 °C, (**D**) 130 °C, and (**E**) 140 °C.

The isothermal measurement of SP-WG blends with a ratio of 7:3 was subjected to high frequency (10 Hz) and high strain (80%) for 15 min in a closed cavity rheometer at high temperature (100–140 °C). The response of these materials with different HSPI content was studied under these conditions to mimic the extrusion process in the high-temperature extrusion. Figure 3 presents the apparent elasticity modulus (G’) of SP-WG blends for variations in heat (100 °C, 110 °C, 120 °C, 130 °C, and 140 °C). Heating the blends from room temperature to the increased temperature caused a reduction in *G*’ at the beginning of the measurement. The G’ of the blend without HSPI increased significantly again and decreased slightly over time at 100 °C, 110 °C, and 120 °C (Figure 3A–C), which was previously explained by the protein’s aggregation or polymerization [7,37,38]. The G’ of SP-WG blends decreased with the increase of HSPI in the measured time range. When HSPI fully replaced the SPI, the trend that the G’ increased over time disappeared at 100 °C, 110 °C, and 120 °C. At 130 °C and 140 °C (Figure 3D,E), the *G’* increased slightly again and immediately decreased over time. The decrease in *G*’ could be due to protein degradation at a higher temperature. Thus, the results indicate that the rheological properties of SP-WG are temperature dependent.

#### 3.2.2. Temperature Sweep of SP-WG Blends

**Figure 4:**
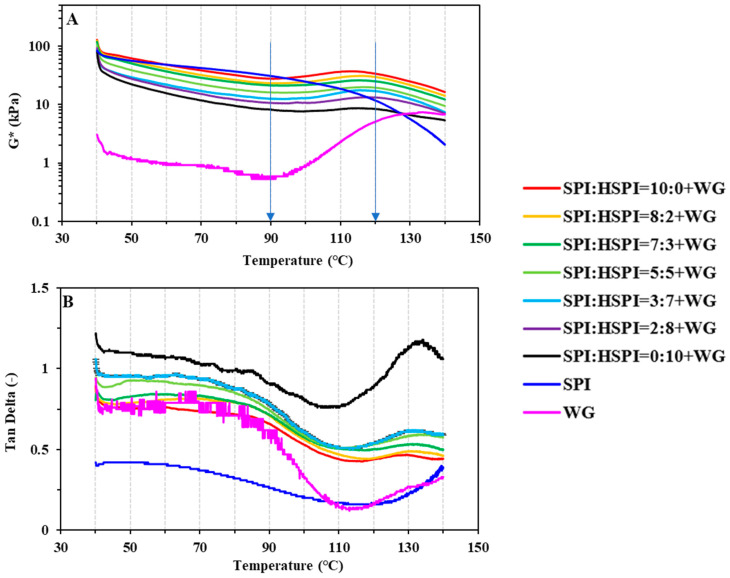
Complex modulus (G*) and tanδ as a function of Soy protein mixture-WG blends, SPI, and WG were shown in (**A**) and (**B**), respectively. From 40 °C to 140 °C, the temperature was increased at a rate of 5 K/min. Constant frequencies and strain (10 Hz and 80%, respectively) were used. The two arrows guide the eye to compare under different temperatures.

Figure 4 shows dynamic rheological changes of SPI, WG, and SP-WG blends for a protein concentration of 45 wt% during heating at 40 °C to 140 °C. The complex modulus (G*) of SPI decreased with increasing temperature; the G* of WG decreased with increasing temperature up to 90 °C and increased again from 90 °C to 140 °C (Figure 4A). For the SP-WG blends, the G* decreased with increasing temperature up to 90 °C, decreased from 90 °C to 110 °C, and decreased again with increasing temperature from 110 °C to 140 °C. The phase shift (tanδ) of WG and SP-WG blends almost kept constant at low temperatures (<90 °C) and sharply decreased from 90 °C to 110 °C (Figure 4B). The initial decrease of G* at low temperatures (<90 °C) can be associated with higher molecular mobility with increasing temperature [39]. The increase of G* and the decrease of tanδ from 90 °C to 110 °C are attributed to an increase in molecular weight due to gluten polymerization reactions [40,41]. The decrease of the G* at high temperatures could be due to the degradation reactions [42]. These rheological results indicated that the WG plays a predominant role in SP-WG blends.

For the SP-WG blends, the G* decreased with increased HSPI within the measured temperature range (Figure 4A) and the G’ decreased with increased HSPI within the measured time range (Figure 3), confirming a plasticizing effect of HSPI. The increased trends in G* of SP-WG blends were weakened with the addition of HSPI, which indicated that the strong hydrophilicity of the small molecule protein in HSPI is detrimental to the WG network formation.

### 3.3. Structural Properties of SP-WG Extrudates

**Figure 5:**
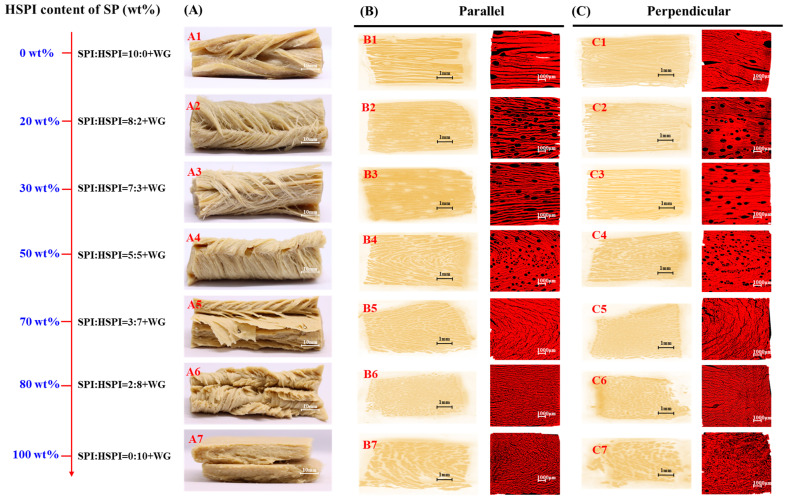
Macrostructure (**A**) and CLSM parallel (**B**) or perpendicular (**C**) to the extrusion direction of the extrudates (SPI:HSPI = 10:0 + WG, SPI:HSPI = 8:2 + WG, SPI:HSPI = 7:3 + WG, SPI:HSPI = 5:5 + WG, SPI:HSPI = 3:7 + WG, SPI:HSPI = 2:8 + WG, and SPI:HSPI = 0:10 + WG).

The macrostructure and CLSM of the SP-WG extrudates in parallel and perpendicular directions are shown in Figure 5. Extrudates’ morphologies were inspected by manually deforming the samples and visually analyzing the structure (Figure 5A). In the absence of HSPI, the extrudates showed coarse fibers (Figure 5A1). The replacement of 20% or 30% SPI with HSPI resulted in structures with evident and slenderer fiber (Figure 5A2,A3). When the HSPI addition was up to 80%, a compact and layered material was obtained (Figure 5A4–A6). When the SP was only HSPI, the extrudate showed a paste and crumbled structure (Figure 5A7). Hagan et al. (1986) reported a similar result that small-molecular-weight peptides from hydrolyzed soy protein could not form a fibrous structure [43].

From the scan images and CLSM, the extrudate without HSPI contained a few voids and appeared to have a loose structure (Figure 5B1,C1). When 20 or 30 wt% SPI was replaced with HSPI, the extrudates had a pronounced fibrous structure (Figure 5B2,B3,C2,C3). These extrudates contained more voids that were less deformed compared with the extrudate without HSPI. When more SPI was replaced with HSPI, the structures of extrudates exhibited more disorder (Figure 5B4–B7,C4–C7). The number and size of voids decreased in these extrudates, which could be related to the reduction of elasticity of SP-WG blends (Figure 4B).

### 3.4. Mechanical Properties of SP-WG Extrudates

**Figure 6:**
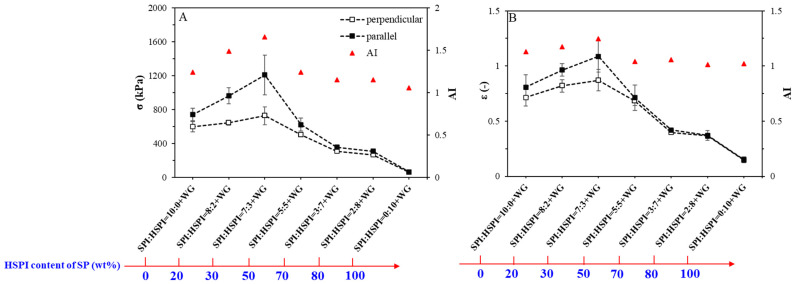
Tensile properties of extrudates: Fracture stress σ (**A**) and fracture strain ε (**B**). The ratio between the averages of the parallel (black bars) and perpendicular directions (white bars) is a measure of anisotropy (AI, red triangles). The dashed lines guide the eye.

The HSPI content affected the macro- and microstructure of SP-WG extrudates, which was expected to influence the mechanical properties. The extrudates were subjected to a tensile test in two directions: in the direction parallel and perpendicular to the extrusion (Figure 6). The fracture stress and strain in both directions increased with the addition of HSPI by up to 30% and then decreased until HSPI entirely replaced the SPI. The anisotropy index of fracture stress and stress increased and decreased with increased HSPI content. When 30% SP was HSPI, the extrudate had the highest anisotropic index of fracture stress (1.7) and strain (1.3). For the extrudates that contained more HSPI (>30%), the anisotropic index of fracture stress or strain was nearly 1, indicating that these extrudates are isotropic. The value of fracture stress and strain decreased with increased HSPI content. A similar result was reported by Seggiani et al. that the fracture stress of the PBSA/HC (Poly Butylene Succinate-co-Adipate/hydrolyzed collagen) blends decreased with increasing HCs, showing a softening effect induced by excess HCs [44]. Therefore, it can be concluded that the HSPI at low concentrations (<30 wt% of SP) can act as a plasticizer to enhance the mechanical anisotropy of SP-WG extrudates.

## 4. Conclusions

Hydrolyzed soy protein isolate (HSPI) was mixed with unhydrolyzed soy protein isolate (SPI) to obtain different soy protein mixtures (SP). These SP were mixed with wheat gluten (WG) at a ratio of 3:7 and extruded with a twin-screw extruder. Adding HSPI at low concentrations (≤30 wt% of SP) promoted fiber formation at macro- and microscales and enhanced mechanical properties. The rheological results showed that the elastic modulus G’ decreased with increased HSPI. The study confirmed that the HSPI containing small molecule protein could act as a plasticizer and, thereby, also influence the viscoelastic properties of the blends during the extrusion process. The extrudates at high HSPI concentration (>30 wt% of SP) became compact and isotropic due to the strong hydrophilicity of the small molecule protein, which is detrimental to the WG network formation. The results from this study suggested that soy protein hydrolysate with a low degree of hydrolyzation can be a potential route for producing meat analogs ingredients in industrial manufacturing practices.

## Data Availability

The data presented in this study are available on request from the corresponding author.
